# Supplementary material for: Relationship between scapular initial position and scapular movement during dynamic motions
Source: PLoS One. 2019 Dec 30;14(12):e0227313. doi: 10.1371/journal.pone.0227313 (PMC6936830; doi:10.1371/journal.pone.0227313)
Supplement: Attachment — Submitted filename: Response to Reviewers.docx [file pone.0227313.s001.docx]

**Response to Reviewer**

Itemized responses to the reviewer’s comments have been listed below in reference to our manuscript entitled “Relationship between scapular initial position and scapular movement during dynamic motions”.

**Reviewer #1**

We thank the reviewer for their constructive comments on our manuscript, as well as for the suggestions provided.

Our responses to your comments are listed below. For clarity, the parts quoted from the manuscript are in red, and the implemented changes are highlighted in yellow.

***Comment 1***

*First, they adopted three motion trackers and secure them to the skin using the tapes. We should carefully consider how accurate they detect the motions. Especially the rotations, the authors demonstrated tiny differences during the motions. It would be helpful for us to understand the outcomes more clearly if the authors could demonstrate the accuracy of the device.*

*In addition, how did they assess the effect of subcutaneous fat on the outcomes. We can assume the authors selected young males without excessive obesity from the overall data. Please mention these parts more carefully at the Materials and Methods.*

**Response**

Thank you for your advice. As you suggested, we have described the device’s accuracy in the manuscript.

Regarding the subjects’ physical appearance, we did not deliberately select subjects that were not obese. On the other hand, we only recruited men because women generally have more subcutaneous fat, which could affect to the sensor, particularly the thoracic sensor. Given this fact we have, therefore, revised the manuscript as follows:

**Revised Materials and methods**

(Page 7, Lines 111-112)

The accuracy of the sensor is 0.03 inches in terms of position and 0.15 degrees in terms of orientation.

(Page 5, Lines 79-80)

Given the measurement error, we also excluded women because they would have more subcutaneous fat than men, which could affect to the sensor, particularly the thoracic sensor.

***Comment 2***

*They described their hyposthesis for this study: the initial position might correlate to the motions. The major problem of this study was the participants’ characteristics. They only assessed “young” “male” subjects. If the authors would like to prove it, I strongly recommend them to assess the participates including elder people and female people as well.*

**Response**

We appreciate your comment. Our results definitely came from a limited population and cannot totally support our hypothesis as you mentioned. Bearing in mind this fact, we have, therefore, revised our hypothesis, purpose, and conclusion by clearly stating the participants’ characteristics. Moreover, the limitation associated with the participants has been included in the discussion section as follows:

**Revised Abstract**

(Page 2, Lines 19-20)

The aim of this study was to discover the relationship between the scapular initial position and scapular movement during dynamic motions in healthy young men.

(Page 2, Lines 29-31)

Except for the internal rotation our results clarified the interactions between the scapular initial position and scapular movement during dynamic motions in healthy young men.

**Revised Introduction**

(Page 4, Lines 68-71)

The aim of this study was to clarify the relationship between scapular initial position and scapular movement during dynamic motions in healthy young men. We hypothesized that there is a positive correlation between the two variables in healthy young men.

**Revised Discussion**

(Page 16, Lines 217-218)

…except that at a few humeral angles in healthy young men.

(Page 16, Line 222)

…flexion and abduction, i.e. dynamic motions, in healthy young men.

(Page 16, Lines 223-224)

…our hypothesis that the scapular initial position is related to scapular movement during dynamic motions in healthy young men.

(Page 20, Lines 292-293)

First, all of the participants were healthy young men, …

(Page 20, Lines 293-294)

…it is not clear whether the findings can be applied to women, older adults, and …

(Page 20, Lines 295-296)

Future studies involving these participants are needed.

**Revised Conclusion**

(Pages 20-21, Lines 308-310)

We investigated the relationship between the scapular initial position and scapular movement in elevation and lowering phase during dynamic motions i.e. flexion and abduction in healthy young men.

***Comment 3***

*The authors assessed three times for each motion. Regarding the methodology, please mention how the authors calculate each motions from triplicated data.*

*In addition, the authors mentioned the reliability of the measurement “the ICC(1,1) calculated from ten healthy men …”. Regarding the reliability, please explain the detail about the values for all motions and rotations, because these information would be helpful to understand the reliability of the methodology using the motion trackers with adhesive tapes.*

**Response**

We have added a sentence to explain the data calculation used from the triplicated scapular measurements.

Additionally, in accordance with your suggestion, we have included details about the scapular measurement reliability. We have changed our minds, and have decided that the intraclass correlation coefficient (1,3) could be more suitable than the intraclass correlation coefficient (1,1) written in the first manuscript version, because we measured the scapular motion three times and the average value was used in further analysis. The reliabilities are presented in Table 1.

**Revised Materials and methods**

(Page 8, Lines 145-146)

The data from the three movements was averaged. The mean value every 30° was then calculated and used for further analysis.

(Page 9, Lines 152-154)

The intraclass correlation coefficient (1,3) calculated from the three scapular movements in ten healthy men [mean age, 25.1 (3.2) years; mean height, 171.1 (6.7) cm; mean weight, 60.5 (13.8) kg] is shown in Table 1, with a high enough reliability.

**Added Table (Page 10)**

**Table 1. Measurement reliability for scapular movements.**

|  | | Elevation phase | | | | Lowering phase | | | |
| --- | --- | --- | --- | --- | --- | --- | --- | --- | --- |
| Elevation angle | | 30° | 60° | 90° | 120° | 120° | 90° | 60° | 30° |
| Flexion | Internal/External rotation | 0.98  (0.96-0.99) | 0.98  (0.95-0.99) | 0.97  (0.91-0.99) | 0.94  (0.83-0.98) | 0.97  (0.92-0.99) | 0.98  (0.94-0.99) | 0.98  (0.95-0.99) | 0.98  (0.94-0.99) |
|  | Downward/Upward rotation | 0.98  (0.96-0.99) | 0.98  (0.94-0.99) | 0.95  (0.86-0.98) | 0.98  (0.95-0.99) | 0.99  (0.97-0.99) | 0.98  (0.94-0.99) | 0.94  (0.82-0.98) | 0.86  (0.61-0.96) |
|  | Posterior/Anterior tilt | 0.97  (0.93-0.99) | 0.98  (0.94-0.99) | 0.98  (0.96-0.99) | 0.98  (0.94-0.99) | 0.98  (0.95-0.99) | 0.97  (0.93-0.99) | 0.96  (0.88-0.98) | 0.91  (0.75-0.97) |
| Abduction | Internal/External rotation | 0.94  (0.83-0.98) | 0.97  (0.93-0.99) | 0.99  (0.98-0.99) | 0.99  (0.97-0.99) | 0.99  (0.97-0.99) | 0.99  (0.98-0.99) | 0.98  (0.96-0.99) | 0.98  (0.95-0.99) |
|  | Downward/Upward rotation | 0.97  (0.92-0.99) | 0.99  (0.97-0.99) | 0.99  (0.98-0.99) | 0.99  (0.98-0.99) | 0.99  (0.98-0.99) | 0.99  (0.98-0.99) | 0.97  (0.92-0.99) | 0.97  (0.93-0.99) |
|  | Posterior/Anterior tilt | 0.99  (0.97-0.99) | 0.99  (0.97-0.99) | 0.98  (0.96-0.99) | 0.99  (0.97-0.99) | 0.99  (0.97-0.99) | 0.99  (0.98-0.99) | 0.97  (0.93-0.99) | 0.99  (0.97-0.99) |

The values are intraclass correlation coefficients (1,3) and the range in parentheses is the 95% confidence interval.

***Comment 4***

*Regarding my understanding, the measurements using the surface motion trackers have disadvantage for the accuracy even they selected limited motion from 30 to 120 degrees. Some researchers used the percutaneous pinning or fluoroscopic measurement to address to the scapula. I suppose that only the upward/downward rotation of the scapula could be assessed with this measurement tool. I recommend the authors to explain the comparison of various techniques to assess the scapular and/or humerus motions with the accuracy values.*

**Response**

Thank you for your comment. Methods based on cutaneous markers are the most popular for recording three-dimensional scapular motion analysis, even though this method includes some measurement errors (Lempereur et al., 2014). Actually, internal/external rotation and anterior/posterior tilt of scapula are evaluated in a large body of literature as well as upward/downward rotation (e.g. Lopes AD et al., 2015; Habechian FA et al., 2014; Braman JP et al., 2009; Teece RM et al., 2008; McClure PW et al., 2001 and so). As you mentioned, on the other hand, it is the fact that subcutaneous tissues such as fat and muscle interfere with accurate measurements of scapular motion in the cutaneous markers method. Therefore, in accordance with your suggestion, we have revised the manuscript by adding previous studies addressing the validity of scapular measurement as follows:

**Revised Discussion**

(Page 20, Lines 296-302)

Second, although we analyzed the scapular movement in humeral elevation up to 120° in the present study, measurement errors could not been completely excluded because the acromial method always included some errors compared with the percutaneous pinning[22] and the tripod measurement[33]. A future study is, therefore, necessary to investigate the precise relationship between the scapular initial position and its movement using 2D/3D registration technique.
