# Supplementary material for: Relationship between scapular initial position and scapular movement during dynamic motions
Source: PLoS One. 2019 Dec 30;14(12):e0227313. doi: 10.1371/journal.pone.0227313 (PMC6936830; doi:10.1371/journal.pone.0227313)
Supplement: Attachment — Submitted filename: Response to Reviewers.docx [file pone.0227313.s002.docx]

**Response to Reviewer**

Itemized responses to the reviewer’s comments have been listed below in reference to our manuscript entitled “Relationship between scapular initial position and scapular movement during dynamic motions”.

**Reviewer #1**

We thank the reviewer for their constructive comments on our manuscript, as well as for the suggestions provided.

Our responses to your comments are listed below. For clarity, the parts quoted from the manuscript are in red, and the implemented changes are highlighted in yellow.

**Comment #1**

Page 7 Lines 111-112

Instead of using inches, I think the authors should use the unit of mm, cm, since they have used cm in the next sentence.

***Response***

*We have changed the unit from inches to mm.*

**Revised manuscript**

The accuracy of the sensor is 0.762 mm …

**Comment #2**

Page 5 Lines 79-80

If the authors described the sentence regarding the exclusion criteria for women, they should demonstrate scientific evidence that women have more measurement error because of the subcutaneous fat.

***Response***

*In accordance with your comment, we have cited a previous study investigating sex differences in skeletal muscle, subcutaneous adipose tissue, bone, and so with use of magnetic resonance imaging* *(Abe et al., 2003). The study included Japanese healthy young men and women as subjects and showed women had higher percentage fat and fat mass than men. This result allows us to assume that women have more measurement error than men because of the subcutaneous fat.*

**Revised manuscript**

…we also excluded women because they would have more subcutaneous fat than men [20], …

**Comment #3**

Again, I recommend the authors to include others with additional characteristics such as elder populations, instead of adding the description of “healthy young men”.

***Response***

*Thank you for your suggestion. You recommend to include population(s) with additional characteristics such as women and the elderly. However, we beg to disagree that this is necessary. Herein there are two reasons.*

1. *Populations with additional characteristics such as older and women, to our knowledge, have a possibility to disturb our result, interpretation and conclusion. Previous studies showed the differences in scapular kinematics between asymptomatic older and young population (Saker, 2014) even when the comparison between men and women (Schwartz et al., 2016). These characteristics dependent on the population are due to several factor. For instance, muscle strength loss is inevitable with age and older people have less muscle strength than young (Larsson et al., 1979; Murray et al., 1985). Anthropometric parameter (Winter, 2009), bone shape (Paraskevas et al., 2008), and neuromuscular function (Anders et al., 2004) are different between gender. Namely, additional population in the current study done not make sense.*
2. *Inclusion of populations such as the elderly and women may create large measurement error between bone and skin due to the subcutaneous tissues. As described in response to your comment#2, women have higher percentage fat and fat mass than men (Abe et al., 2003) and older groups show thicker fat than young groups (Kanehisa et al., 2004).*

*Bearing in mind these difficulties, we decided to limit the population to healthy young men. As described in our limitations, it is not clear whether the findings can be applied to women, older adults, and individuals with shoulder disorders. However, the lack of inclusion of other populations does not imply that our data do not have scientific value. In the future we plan to conduct these experiment including populations such as older adults and women. In short, we believe that excluding such populations does not make our results invalid.*

*Since this is the second time you have raised this point, we understand that you feel it is important. However, you have not provided any scientific rationale to convince us of that importance. Moreover, thousands of studies have been published which consider just one population. Population comparisons is therefore clearly not a prerequisite for publication in general. While we agree that a population comparison would be useful, and would also be necessary to demonstrate general validity, we believe that these are separate issues that are tangential to our paper’s message.*

*If you still feel that it is necessary to add additional populations, we respectfully request that you clarify the scientific rationale for your recommendation.*

**Comment #4**

They modified the sentence regarding the limitation. I suppose they should have descripted how they justified the internal/external rotation as well as anterior/posterior tilt with scientific values in advance to this study.

***Response***

*We apologize, but we do not understand this comment. Please allow us to rephrase your comment as follows: “Authors should use a priori rational to explain why they used internal/external rotation and anterior/posterior tilt.”*

*If this is what your comment means, we believe that our Introduction and Methods already sufficiently detail the rationale for choosing these tasks.*

*If this is not what your comment means, we respectfully request clarification.*
